# Financial incentives improve recognition but not treatment of cardiovascular risk factors in severe mental illness

**DOI:** 10.1371/journal.pone.0179392

**Published:** 2017-06-09

**Authors:** Carol L. Wilson, Kirsty M. Rhodes, Rupert A. Payne

**Affiliations:** 1 Department of Public Health and Primary Care, University of Cambridge, Cambridge, United Kingdom; 2 MRC Biostatistics Unit, Cambridge, United Kingdom; 3 Centre for Academic Primary Care, School of Social and Community Medicine, University of Bristol, Bristol, United Kingdom; Institute of Psychiatry, UNITED KINGDOM

## Abstract

**Objectives:**

Severe mental illness (SMI) is associated with premature cardiovascular disease, prompting the UK primary care payment-for-performance system (Quality and Outcomes Framework, QOF) to incentivise annual physical health reviews. This study aimed to assess the QOF’s impact on detection and treatment of cardiovascular risk factors in people with SMI.

**Methods:**

A retrospective open cohort study of UK general practice was conducted between 1996 and 2014, using segmented logistic regression with 2004 and 2011 as break points, reflecting the introduction of relevant QOF incentives in these years. 67239 SMI cases and 359951 randomly-selected unmatched controls were extracted from the Clinical Practice Research Datalink (CPRD).

**Results:**

There was strong evidence (p≤0.015) the 2004 QOF indicator (general health) resulted in an immediate increase in recording of elevated cholesterol (odds ratio 1.37 (95% confidence interval 1.24 to 1.51)); obesity (OR 1.21 (1.06 to 1.38)); and hypertension (OR 1.19 (1.04 to 1.38)) in the SMI group compared with the control group, which was sustained in subsequent years. Similar findings were found for diabetes, although the evidence was weaker (p = 0.059; OR 1.21 (0.99 to 1.49)). There was evidence (p<0.001) of a further, but unsustained, increase in recording of elevated cholesterol and obesity in the SMI group following the 2011 QOF indicator (cardiovascular specific). There was no clear evidence that the QOF indicators affected the prescribing of lipid modifying medications or anti-diabetic medications.

**Conclusion:**

Incentivising general physical health review for SMI improves identification of cardiovascular risk factors, although the additional value of specifically incentivising cardiovascular risk factor assessment is unclear. However, incentives do not affect pharmacological management of these risks.

## Introduction

People with severe mental illnesses (SMI), including schizophrenia and bipolar affective disorder, are known to be at significant risk of premature morbidity and mortality. In the UK, individuals with SMI have a life expectancy around 12 years less than the general UK population,[1] and similar disparities are seen in the US.[2] Cardiovascular disease is a major contributor to this health inequality.[3]

The UK Quality and Outcomes Framework (QOF) aims to improve quality in primary care through linking financial incentives to performance against indicators.[4] From its inception in 2004, the QOF has incentivised annual physical health review for people with SMI (S1 Appendix). The nature of this review was unspecified until 2011, when more explicit cardiovascular risk factor indicators were introduced; the majority of indicators were withdrawn in 2014.

To date there has been little evaluation of the impact of the QOF on the recognition and management of cardiovascular risk factors in people with SMI. One study found the QOF incentives reduced inequalities in cardiovascular risk factor testing between those with and without SMI,[5] although the nature and time period of the analysis was relatively limited and did not evaluate risk factor detection. Elsewhere the QOF has been shown to have increased consultation rates[6] in people with SMI and also to have coincided temporally with an increase in recording of comorbidities[6,7].

The current study builds on the above findings by exploring whether the QOF indicators have been associated with improvements in the identification and management of cardiovascular risk factors in people with SMI.

## Methods

Anonymised data detailing diagnoses, prescribing, test results and demographics were extracted from the Clinical Practice Research Datalink (CPRD). CPRD captures data recorded by the general practitioner (GP) as part of routine care, and covers a 6.9% representative sample of the UK population.[8] Patients were included in the analysis during continuous periods of registration at those participating practices which met CPRD’s internal quality standards. The study was approved by the CPRD Independent Scientific Advisory Committee (protocol reference 15_110RMn).

### Study design

A retrospective open cohort design was used with cases having a lifetime SMI diagnosis and an unmatched population comparison group without SMI. The study period included the consecutive 'financial years' from 1st April 1995 to 31st March 2014. Two interventions were considered: intervention 1 from April 2004 (introduction of the QOF SMI annual review indicator), and intervention 2 from April 2011 (change to specific cardiovascular indicators).

Data from April 1995 to March 2003 were used to ascertain trends in the outcome before the introduction of QOF. The 2003/04 year was excluded as per previous studies[6], as some practices were preparing for the introduction of the QOF in the year prior to its introduction.

Within each year of analysis case and comparison group members could be eligible for the full financial year or for a proportion of the year. The first day of inclusion in the analysis was the latest of the patient reaching age 35, first day of continuous registration, the patient's GP practice meeting CPRD’s data quality criteria, or (for cases) the date of SMI diagnosis. Eligibility for inclusion in the analysis ended at the first of having the outcome of interest, leaving the practice, death, or the practice ceasing to submit data to CPRD.

### Case and comparison group

Cases included all available patients aged ≥35 years with a life-time diagnosis of SMI. SMI was defined as schizophrenia, bipolar affective disorder, psychotic depression and other non-transient, non-organic psychoses. We identified Read codes corresponding to these conditions. Code lists were based on previously published lists from the clinicalcodes.org repository[9], supplemented by free-text searches for the aforementioned clinical terms. A snowballing approach was then employed to identify additional terms similarly categorised in the Read code hierarchy. Additional QOF codes were included to capture any change in coding practice post-QOF. The resulting list of codes was reviewed by two clinicians to confirm the appropriateness or otherwise of included codes, as well as identifying excluded diagnoses. As way of validation, the prevalence of the resulting outcomes was checked against existing literature and alternative published code lists. Patients with codes related to prodromal schizophrenia, to a “single episode” or to a “reactive” episode were excluded, unless they also had a valid diagnostic SMI code recorded. Code lists are available from the University of Bristol Research Data Repository.[10]

The comparison group consisted of unmatched, randomly selected patients aged ≥35 years without SMI who had a period of continuous registration during the study period, aiming for a minimum ratio of controls to cases of 5:1. Patients were excluded if they had ever been prescribed medication used in the treatment of SMI, with the exception of those who had ever had an epilepsy Read code recorded (i.e. when there is a likelihood of medication being used as an anticonvulsant rather than a mood stabiliser).

We placed a restriction on the lower age limit of our population as outcomes are rare in those under 35 years, and this improved our power to detect differences.

### Clinical outcomes

We assessed four outcomes related to diagnosis and two related to treatment, which we considered of clinical importance and straightforward to measure using routine health-record data: first ever recording of elevated serum cholesterol ≥5.0mmol/L, first ever diagnosis of diabetes mellitus, first ever diagnosis/recording of obesity, first ever diagnosis of hypertension, first ever prescription of anti-diabetic medication, and first ever prescription of lipid-modifying medication.

Hypertension and diabetes mellitus outcomes were identified by diagnostic and administrative Read codes, elevated cholesterol by test results, obesity by Read codes or body mass index values ≥30.0kg/m^2^, and medications from product code lists identified from the CPRD dictionary. A similar approach to code list development was employed as for the identification of SMI.

### Statistical analyses

Annual incidence rates were calculated for all six outcomes for the case and comparison groups. The proportion of the financial year spent eligible for inclusion in the analysis by each patient was combined to create a denominator of 'person-years' active for each financial year for both groups.

For all outcomes an interrupted time series analysis (ITSA) was performed. This approach is recognised to be amongst the strongest quasi-experimental approaches to intervention analysis.[11,12] ITSA utilises data collected over equally spaced time intervals before and after an intervention. It assumes that, in the absence of the intervention, trends prior to the intervention could have been extrapolated to predict future trends.

A mixed effect segmented logistic regression model was used for the ITSA, with the inclusion of a random intercept to allow for variation between general practices. The ITSA model has been described elsewhere[13] and can be extended to incorporate a control group, as shown in S2 Appendix. Additional terms were added to allow analysis of the second intervention (change to QOF SMI indicators in 2011) and potential confounding by age (categorised using 40, 50 and 60 year cut-offs) and gender. Data manipulation was undertaken using *Stata13*.*0*[14] and analyses were conducted in *R (version 3*.*2*.*4)* using the package *lme4*.[15] Step changes occurring immediately after the intervention’s introduction are reflected in the model intercept, with subsequent effects on the temporal trend reflected by the model slope. Model fit was assessed by plotting the predicted probability of the outcome from the fitted model against the observed probability of the outcome for the SMI and non-SMI groups (S3 Appendix).

We hypothesised that outcome measurements closer together in time may be more similar than outcomes further apart. We used the Cumby-Huizinga test to explore the data for the presence of this issue (autocorrelation), which may have resulted in part due to the grouping of patients by general practice. The autocorrelation was almost zero and appeared to be negligible (the test for lag orders 1 to 5 strongly accepted the null hypothesis of independence in the series, as did the test at the individual lag). On account of the size of the dataset and complexity of the statistical models, we therefore decided to take a parsimonious approach and not account for potential autocorrelation in subsequent analyses.

## Results

The number of patients and practices included varied with the growth of the CPRD dataset, with 232, 595 and 530 practices contributing data in 1995/96, 2004/05 and 2013/14 respectively. Numbers of patients and demographic characteristics are summarised in Table 1. A total of 67,239 people with SMI and 359,951 people without SMI were included in the analysis, with the former group typically providing fewer days of continuous data (1936 vs. 2703 days) in part due to increased mortality.

**Table 1:** Demographic characteristics of patients with and without severe mental illness.

	Year	SMI	Non-SMI
Number of patients	1995/96	6,395	59,476
	2004/05	19,728	179,434
	2013/14	23,775	174,306
Age in years, mean (SD)	1995/96	63.0 (SD 16.3)	56.0 (SD 14.6)
	2004/05	57.6 (SD 14.9)	55.6 (SD 14.6)
	2013/14	56.8 (SD 14.3)	56.7 (SD 14.5)
Male	1995/96	38.4%	48.9%
	2004/05	45.2%	49.3%
	2013/14	48.4%	49.0%
Socioeconomic deprivation (IMD quintile)			
1 (least deprived)	-	9.6%	15.0%
2		11.6%	15.0%
3		12.6%	12.8%
4		14.4%	11.7%
5 (most deprived)		15.0%	8.9%
Unavailable		36.8%	36.7%
Follow-up in days, mean (SD)	-	1936 (SD 1785)	2703 (SD 2051)

SMI, severe mental illness; SD, standard deviation; IMD, Index of Multiple Deprivation. IMD quintile based on the IMD score for relevant postcode in 2010 (unavailable for over a third of patients, including all patients outside of England)

**Fig 1:**
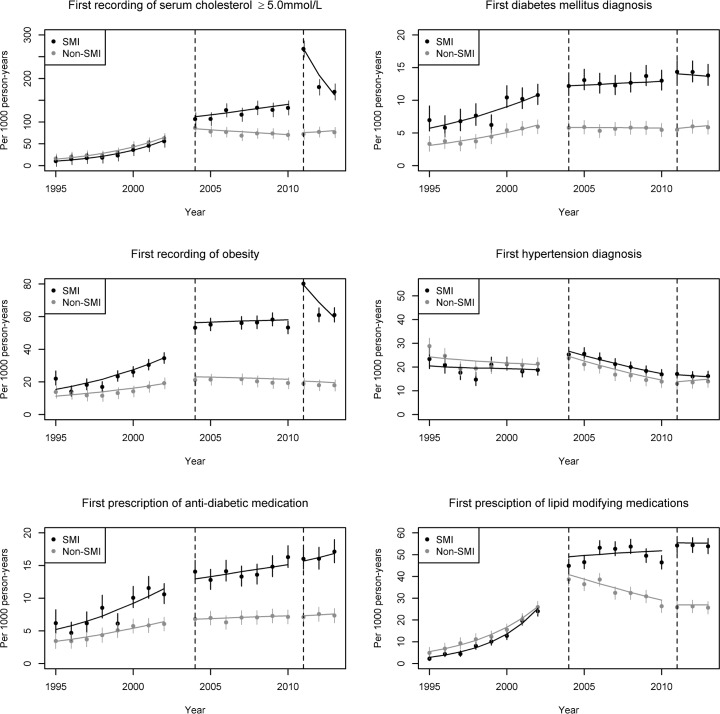
Outcomes over time for patients with and without severe mental illness. SMI, severe mental illness. Dashed vertical lines indicate the introductions of the Quality of Outcomes Framework (QOF) in 2004 and 2011.

Fig 1 shows the ITSA results. Numerical results are reported in Table 2 (detection of risk factors) and Table 3 (treatment of risk factors). Following the incentivisation of annual reviews for people with SMI in April 2004, there is strong evidence of an immediate increase (i.e. intercept change) in the recording of elevated cholesterol.

**Table 2:** Interrupted time series analyses results, reporting immediate changes in detection of cardiovascular risk factors and changes over time.

		Odds ratio (95% confidence interval)			
		SMI	Non-SMI	Difference	p value*
Serum cholesterol ≥ 5.0mmol/L					
Intervention 1	Change in intercept	1.21 (1.10 to 1.33)	0.89 (0.86 to 0.92)	1.37 (1.24 to 1.51)	<0.001
	Change over time	0.80 (0.78 to 0.82)	0.78 (0.77 to 0.78)	1.03 (1.00 to 1.05)	0.030
Intervention 2	Change in intercept	1.95 (1.83 to 2.08)	1.08 (1.04 to 1.11)	1.84 (1.72 to 1.97)	<0.001
	Change over time	0.70 (0.67 to 0.73)	1.04 (1.02 to 1.06)	0.67 (0.65 to 0.70)	<0.001
Diabetes mellitus					
Intervention 1	Change in intercept	0.94 (0.78 to 1.13)	0.78 (0.71 to 0.85)	1.21 (0.99 to 1.49)	0.059
	Change over time	0.90 (0.87 to 0.94)	0.88 (0.86 to 0.90)	1.02 (0.98 to 1.09)	0.286
Intervention 2	Change in intercept	1.08 (0.93 to 1.24)	1.00 (0.92 to 1.09)	1.08 (0.91 to 1.27)	0.391
	Change over time	0.96 (0.88 to 1.04)	1.02 (0.97 to 1.07)	0.94 (0.85 to 1.03)	0.191
Obesity					
Intervention 1	Change in intercept	1.30 (1.15 to 1.46)	1.07 (1.02 to 1.13)	1.21 (1.06 to 1.38)	0.005
	Change over time	0.88 (0.86 to 0.91)	0.91 (0.90 to 0.92)	0.98 (0.95 to 1.00)	0.087
Intervention 2	Change in intercept	1.35 (1.24 to 1.47)	0.97 (0.92 to 1.02)	1.39 (1.26 to 1.53)	<0.001
	Change over time	0.85 (0.80 to 0.89)	0.98 (0.95 to 1.01)	0.87 (0.82 to 0.92)	<0.001
Hypertension					
Intervention 1	Change in intercept	1.47 (1.28 to 1.68)	1.23 (1.17 to 1.29)	1.19 (1.04 to 1.38)	0.015
	Change over time	0.93 (0.90 to 0.96)	0.91 (0.90 to 0.92)	1.02 (0.99 to 1.05)	0.240
Intervention 2	Change in intercept	1.03 (0.90 to 1.17)	1.04 (0.98 to 1.10)	0.99 (0.85 to 1.14)	0.849
	Change over time	1.02 (0.94 to 1.11)	1.11 (1.07 to 1.15)	0.92 (0.84 to 1.00)	0.055

Changes are reported for patients with and without severe mental illness, and the difference between the two groups. SMI, severe mental illness.

* p value for difference between groups

**Table 3:** Interrupted time series analyses results, reporting immediate changes in treatment of cardiovascular risk factors and changes over time.

		Odds ratio (95% confidence interval)			
		SMI	Non-SMI	Difference	p value*
Anti-diabetic medication					
Intervention 1	Change in intercept	0.90 (0.75 to 1.07)	0.91 (0.84 to 0.99)	0.98 (0.81 to 1.20)	0.864
	Change over time	0.90 (0.87 to 0.94)	0.91 (0.89 to 0.93)	0.99 (0.95 to 1.043	0.587
Intervention 2	Change in intercept	1.01 (0.88 to 1.16)	1.00 (0.93 to 1.08)	0.98 (0.94 to 1.03)	0.423
	Change over time	0.99 (0.91 to 1.07)	0.98 (0.94 to 1.03)	1.01 (0.92 to 1.10)	0.906
Lipid-modifying medications (including statins)					
Intervention 1	Change in intercept	1.06 (0.93 to 1.22)	1.06 (1.01 to 1.11)	1.01 (0.987 to 1.16)	0.924
	Change over time	0.73 (0.71 to 0.76)	0.75 (0.74 to 0.76)	0.98 (0.94 to 1.01)	0.187
Intervention 2	Change in intercept	1.05 (0.97 to 1.15)	0.97 (0.93 to 1.01)	1.10 (1.00 to 1.20)	0.053
	Change over time	0.96 (0.92 to 1.01)	1.03 (1.00 to 1.06)	0.94 (0.89 to 0.99)	0.027

Changes are reported for patients with and without severe mental illness, and the difference between the two groups. SMI, severe mental illness.

* p value for difference between groups

Immediately after the intervention in 2004, the odds of an SMI patient having elevated cholesterol test results are 1.21 (95% CI: 1.10–1.33) times higher, after the intervention in 2004. These odds are 37% (95% CI: 24%-51%) higher than for a non-SMI patient. The results similarly show increased recognition of diabetes (OR 1.21, 95% CI: 0.99–1.49), obesity (OR 1.21, 95% CI: 1.06–1.38) and hypertension (OR 1.19, 95% CI: 1.04–1.38), in the SMI compared to the non-SMI group (Table 2).

A relative change over time coinciding with the 2004 QOF incentives was seen only for elevated cholesterol (OR 1.03, 95% CI: 1.00–1.05), whereas the remainder saw no relative change in gradient from 2004 to 2010, suggesting the immediate effects observed were sustained.

The introduction of cardiovascular specific SMI indicators in 2011 was associated with further immediate increases (i.e. changes in intercept) in recognition of cases of elevated cholesterol (OR 1.84, 95% CI: 1.72–1.97) and obesity (OR 1.39, 95% CI: 1.26–1.53) relative to the non-SMI group. However, this increase was not sustained over the following two years that the incentives remained in place, with significant reducing trends relative to the non-SMI group. No added improvements in case-finding of diabetes or hypertension were found in association with the 2011 incentives.

Prescribing of both anti-diabetic and lipid-modifying medications increased significantly over the study period, and to a greater extent in the SMI group. There was no evidence that these changes were related to the QOF indicators introduced in 2004 (Table 3). The SMI indicator introduced in 2011 was also not found to be associated with an increase in prescribing of anti-diabetic medications in the SMI group relative to the non-SMI group.

Following the 2011 QOF SMI incentives, there was also no strong evidence of an immediate change in prescribing of lipid modifying medications. There was some indication that the change in the effect of time attributable to the 2011 QOF indicator differed for the SMI group compared to the non-SMI group (OR 0.94, 95% CI: 0.89–0.99).

In the early years of the study there was a second peak in age of diagnosis of SMI in older patients (≥60 years) which was absent following the introduction of QOF, raising concerns that a change in coding practice had influenced the findings. As a sensitivity analysis, we removed patients whose age at first diagnosis of SMI was at least 60 years; this had little impact on our findings (S4 Appendix). In addition, we conducted a falsification analysis, using fabricated policy indicator years of 2000 and 2008 (S5 Appendix). There were no significant changes in outcome observed in relation to these dates. The only exception was an apparent change in recording of elevated cholesterol in 2008 but this was in the opposite direction to that observed in the main 2011 analysis. These findings provide reassurance that the observed changes in outcomes in the main analysis are indeed related to the QOF interventions.

## Discussion

We have found strong evidence that primary care incentives promoting physical health reviews in patients with SMI can result in improvements in the identification but not treatment of cardiovascular risk factors.

This study benefits from the use of robust methodology and external generalizability. Interrupted time series analysis with a comparison group is a powerful, pseudo-experimental methodology that allows both the temporal trends prior to the intervention and any change coinciding with the intervention to be taken into account. However, the study’s limitations also require consideration. First, it is not possible to distinguish improved case finding from genuine changes in incidence, although the latter are likely to happen gradually. It is also possible that the increases in diagnoses reflect improved recording as opposed to detection *per se*. Nevertheless, we believe that recording of clinical information in electronic health records is still likely to facilitate delivery of care, such as through improved case finding, and thus failure to record a risk factor is for all practical purposes equivalent to failing to identify it in the first place. In addition, laboratory values (e.g. cholesterol) are automatically populated from the laboratory systems, and as such reflect genuine measurement and not simply recording. Secondly, we found differences in the age distribution of our SMI population in the earlier years of the study, potentially reflecting changes in coding practice; a fall over time in the age at diagnosis of SMI has also been described elsewhere.[6] Nevertheless, we adjusted for age in our analyses, and also found no notable differences when age was not accounted for by the models, reassuring us that our findings are robust to this issue. Additional supplementary analyses, excluding those with a first diagnosis of SMI at age 60 years or older, supports these findings. Thirdly, ITSA may be rendered invalid by the presence of a confounder that changed in a time period coinciding with the intervention. This is pertinent to the current study as the QOF targeted other broad key health outcomes in the general population, some of which may also have been relevant to people with SMI. This is accounted for by the inclusion of a comparison group in the study design, although the assumption must be made that other health service changes would impact on both groups equally. We are unaware of other major interventions (e.g. clinical guidelines) that coincided temporally with the QOF changes. Although National Service Frameworks were introduced in the early 2000s to set minimum standards for care in areas including diabetes, we expect that the impact of this is likely to be small, as firstly our study focuses on case identification rather than quality of care, and secondly there is no reason to suspect it to have a significant differential impact on the two study groups. Fourthly, the QOF SMI indicators introduced in 2011 relating to lipids and blood glucose/HbA1c testing applied only to those aged 40 and over whilst cases aged 35 and over are included in this study. This could have resulted in a slight underestimate of the true effect. Finally, the study is limited by the small number of data points following the 2011 changes to the QOF SMI indicators. As these indicators were in place for only 3 years, a more complete analysis including longer term trends is impossible, although we believe our analysis probably still provides valid and useful insights into the effect of the later intervention.

Under-recording of cardiovascular risk factors in the SMI population has been previously reported[16] and it is thus reassuring that incentives appear to help address this. When specific cardiovascular SMI indicators were introduced in 2011, people with SMI had already been subject to annual reviews for seven years, meaning those most willing to attend reviews who were at highest cardiovascular risk may already have been identified. This contextual difference makes it difficult to know whether there are differences in the effectiveness of the 2004 and 2011 indicators. Of note, the sharp increases in recording of both obesity and elevated cholesterol in 2011 despite the seven preceding years of annual reviews perhaps suggest the later cardiovascular-specific indicator resulted in a “catch-up” of previously undetected risk factors; the subsequent drop in recording may reflect patients being tested in earlier years or being otherwise harder to reach. Despite this, the levels of detection do not fall below the pre-2011 trend for either elevated cholesterol or obesity. One must therefore be cautious about both interpreting the initial improvement in 2011 as evidence of the superiority of a more specific incentive, and of interpreting the post-2011 decrease as meaning this indicator was either not sustained or less effective than the indicators introduced in 2004. The majority of indicators were withdrawn in 2014 meaning a more complete analysis of the 2011 indicators will not be possible: it is not clear whether they were in place for sufficiently long to become usual practice.

The analysis suggests that the QOF SMI indicators have not affected prescribing of either anti-diabetic or lipid-modifying medications. This is despite longstanding (i.e. pre-QOF), readily available UK clinical guidance on pharmacotherapy for the associated risk factors in the general population.[17] National guidance[18] advising clinicians to be aware of and treat increased cardiovascular risk in SMI (specifically schizophrenia) patients has only been available since 2009, but further work is nonetheless required to explore the reasons why the increase in detection of risk factors is not matched by increased treatment. The findings are consistent with studies describing under-treatment in this population in the presence of dyslipidaemia and hyperglycaemia[19] and in other areas such as stroke[20] and arthritis[21]. A number of potential explanations for this have been proposed, including at the patient-level (e.g. cognitive impairment, poor adherence), physician-level (e.g. stigmatization, complexity of care), and service-level (e.g. fragmentation of care, lack of resources).[22] It is unclear whether the increase in medication use due to the QOF prescribing incentives that has been observed in the general population[23] would translate to an increase in prescribing if targeted specifically at the SMI population.

A number of issues raised by this study merit further investigation. These include whether or not more specific cardiovascular indicators offer additional value, patients’ and clinicians’ views on the role of SMI indicators, the reason that case detection was not followed by improvements in treatment, and whether there is value in specific incentives to encourage treatment of physical problems in this population. However, what remains clear is that financial incentives for GPs improve the detection of cardiovascular risk factors in a challenging patient group in which identification of physical health problems is known to be poor. Incentives may well have a broader role in reducing health inequalities and improving the care and treatment of patients with severe mental illness.

## Supporting information

S1 Appendix Quality and Outcomes Framework (QOF) indicators relevant to severe mental illness. (DOCX)

S2 Appendix Regression model. (DOCX)

S3 Appendix Assessment of model fit. (DOCX)

S4 Appendix Sensitivity analyses with patients diagnosed with severe mental illness aged 60 years and over removed. (DOCX)

S5 Appendix Falsification test. (DOCX)
