# Supplementary material for: Financial incentives improve recognition but not treatment of cardiovascular risk factors in severe mental illness
Source: PLoS One. 2017 Jun 9;12(6):e0179392. doi: 10.1371/journal.pone.0179392 (PMC5466340; doi:10.1371/journal.pone.0179392)
Supplement: S1 Appendix — (DOCX) [file pone.0179392.s001.docx]

**Appendix S1. Quality and Outcomes Framework (QOF) indicators relevant to severe mental illness.**

|  |  | Year | Indicator | Description |
| --- | --- | --- | --- | --- |
|  |  |  |  |  |
| Intervention 1 |  | 2004/05 to 2005/06 | MH1 | Register of patients with severe long-term mental health problems |
|  |  |  | MH2 | Percentage of MH1 with review recorded within 15 months (included check on accuracy of prescribed medication, a review of physical health, and a review of coordination arrangements with secondary care) |
|  |  | 2006/07 to 2010/11 | MH8 | Register of patients with schizophrenia, bipolar disorder and other psychoses (replaces MH1) |
|  |  |  | MH9 | Percentage of MH8 with a review recorded in the preceding 15 months, including routine health promotion and prevention advice appropriate to their age, gender and physical health status (replaces MH2) |
|  |  |  |  |  |
| Intervention 2 |  | 2011/12 | MH8 | Register of patients with schizophrenia, bipolar disorder and other psychoses |
|  |  |  | MH12 | Percentage of MH8 with record of BMI in preceding 15 months (replaces MH2) |
|  |  |  | MH13 | Percentage of MH8 with record of blood pressure in preceding 15 months (replaces MH2) |
|  |  |  | MH14 | Percentage of MH8 with record of total:HDL cholesterol ratio in preceding 15 months (aged 40 years and over only) (replaces MH2) |
|  |  |  | MH15 | Percentage of MH8 with record of blood glucose in preceding 15 months (aged 40 years and over only) (replaces MH2) |
|  |  | 2012/13 | MH8 | Register of patients with schizophrenia, bipolar disorder and other psychoses |
|  |  |  | MH12 | Percentage of MH8 with record of BMI in preceding 15 months |
|  |  |  | MH13 | Percentage of MH8 with record of blood pressure in preceding 15 months |
|  |  |  | MH19 | Equivalent to MH14 |
|  |  |  | MH20 | Equivalent to MH15 except HbA_1c_ can be used as alternative to blood glucose |
|  |  | 2013/14 | MH001 | Register of patients with schizophrenia, bipolar disorder and other psychoses and other patients on lithium therapy (replaces MH8) |
|  |  |  | MH003 | Equivalent to MH13, except time limit reduced from 15 to 12 months |
|  |  |  | MH004 | Equivalent to MH14, except time limit reduced from 15 to 12 months |
|  |  |  | MH005 | Equivalent to MH15, except time limit reduced from 15 to 12 months |
|  |  |  | MH006 | Equivalent to MH12, except time limit reduced from 15 to 12 months |
|  |  |  |  |  |

Table shows the relevant QOF indicators relating to severe mental illness and cardiovascular disease risk. From 2004/05 to 2010/11 there was little change, with annual reviews including a focus on physical health, but the choice of any tests performed was left to the discretion of the clinician completing the review. This period is termed as “intervention 1” in the analyses. From 2011/12 to 2013/14 specific cardiovascular indicators were included. This period is termed “intervention 2” in the analyses.
