# Supplementary material for: Financial incentives improve recognition but not treatment of cardiovascular risk factors in severe mental illness
Source: PLoS One. 2017 Jun 9;12(6):e0179392. doi: 10.1371/journal.pone.0179392 (PMC5466340; doi:10.1371/journal.pone.0179392)
Supplement: S2 Appendix — (DOCX) [file pone.0179392.s002.docx]

**Appendix S2. Regression model.**

Mixed effects logistic regression model to investigate the influence of a single intervention on a binary outcome in cases of severe mental illness compared with controls.

Equation

**logit(μ_ij_) = β_0_ + β_1_ × time_ij_ + β_2_ × intervention_ij_ + β_3_ × time_after_intervention_ij_ + β_4_ × case_ij_ +**

**β_5_ (case_ij_ × time_ji_) + β_6_(case_ij_ × intervention_ij_) + β_7_ (case_ij_ × time_after_intervention_ij_) + b_j_**

Observations indexed *i* within practices *j*

| **μ_t_** | the probability that the binary outcome variable equals 1 |
| --- | --- |
| **time** | the number of units of time from the start of the study |
| **intervention** | takes the values 0 in the pre-intervention segment and 1 in the post- intervention segment |
| **time_after_intervention** | interaction between time and intervention, which takes the value 0 in the pre-intervention segment and counts the units of time in the post-intervention segment at time *t* |
| **case** | indicator of a case of severe mental illness |
| **β_0_** | estimates the base level of the outcome at the beginning of the series |
| **β_1_** | estimates the base trend, i.e. the change in outcome per unit time, in the pre-intervention segment |
| **β_2_** | estimates the change in level in the post-intervention segment |
| **β_3_** | estimates the change in trend in the post-intervention segment |
| **ß_4_** | represents the difference in the level (intercept) of the outcome between the case and control groups prior to the intervention |
| **ß_5_** | represents the difference in the trend (slope) of the outcome variable between the case and control groups prior to the intervention |
| **ß_6_** | indicates the difference between the change in level in the case and control groups in the period in which the intervention was introduced |
| **ß_7_** | represents the difference between the change in the slope (trend) the case and control groups in following the introduction of the intervention |
| **b_j_** | random intercept for practice *j,* assumed to have a normal distribution with mean 0 and variance τ^2^, where τ^2^ estimates the between-practice variance in intercept |
