# Supplementary material for: Financial incentives improve recognition but not treatment of cardiovascular risk factors in severe mental illness
Source: PLoS One. 2017 Jun 9;12(6):e0179392. doi: 10.1371/journal.pone.0179392 (PMC5466340; doi:10.1371/journal.pone.0179392)
Supplement: S3 Appendix — (DOCX) [file pone.0179392.s003.docx]

**Appendix S3. Assessment of model fit.**

**
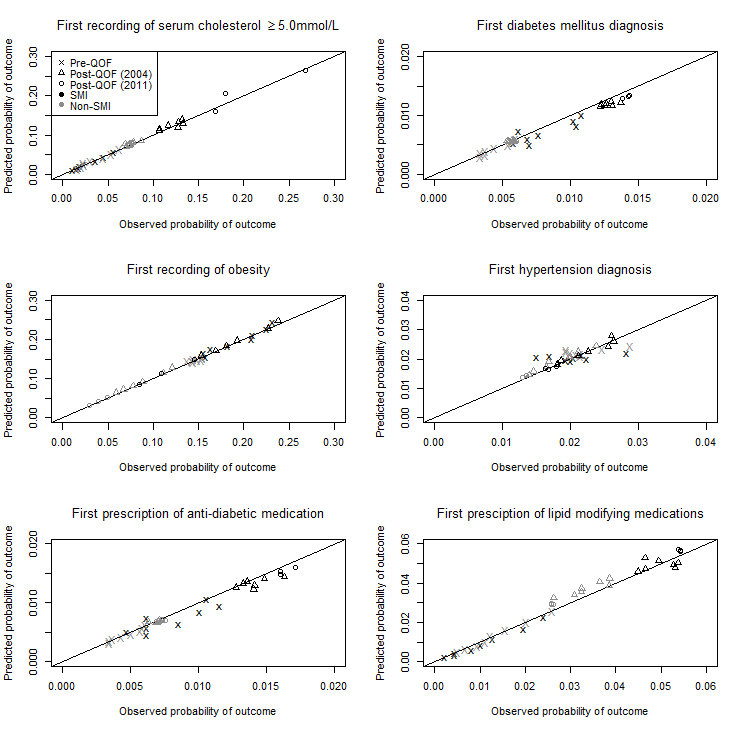
**Plots of the predicted probability vs the observed probability of each outcome for the SMI and non-SMI groups. The relationship is reasonably close to a straight line, and the models appear to fit the data well.

SMI, Severe Mental Illness; QOF, Quality and Outcomes Framework.
