# Supplementary material for: Financial incentives improve recognition but not treatment of cardiovascular risk factors in severe mental illness
Source: PLoS One. 2017 Jun 9;12(6):e0179392. doi: 10.1371/journal.pone.0179392 (PMC5466340; doi:10.1371/journal.pone.0179392)
Supplement: S4 Appendix — (DOCX) [file pone.0179392.s004.docx]

**Appendix S4. Sensitivity analyses with patients diagnosed with Severe Mental Illness aged 60 years and over removed.**

The mean age of the SMI group (cases) fell significantly over time. Further exploration of the data shows that in the first years of the analysis there is a second peak in diagnosis in later life which is much reduced by 2004/05 and completely absent by the most recent years (see Figure S4 below). This finding may relate to a change in coding practices; in the earlier years, it is possible that psychotic symptoms in the context of dementia were coded using codes which were later used only for people on SMI registers. The implication of this is that some of the 'cases' may not have a true diagnosis of SMI. In order to explore the impact of this we carried out a sensitivity analysis with all people whose age at first 'SMI diagnosis' was 60 years or more removed. These results are presented in Table S4 below. In this analysis the mean age of the SMI group was 54.2 in 1995/1996, 53.7 in 2004/2005 and 53.9 in 2013/2014.

*Figure S4. Population age density plots over time*

*Table S4. Interrupted time series analysis model results excluding patients diagnosed with SMI aged 60 years and over*

|  | | Odds ratio (95% confidence interval) | | |  |
| --- | --- | --- | --- | --- | --- |
|  | | SMI | Non-SMI | Difference | p value* |
| **Serum cholesterol ≥ 5.0mmol/L** | | | | | |
| Intervention 1 | Change in intercept | 1.24 (1.13 to 1.37) | 0.87 (0.85 to 0.90) | 1.42 (1.28 to 1.58) | <0.001 |
|  | Change over time | 0.84 (0.82 to 0.86) | 0.79 (0.78 to 0.80) | 1.07 (1.04 to 1.10) | <0.001 |
| Intervention 2 | Change in intercept | 1.79 (1.69 to 1.90) | 1.07 (1.04 to 1.11) | 1.67 (1.56 to 1.78) | <0.001 |
|  | Change over time | 0.75 (0.72 to 0.78) | 1.05 (1.04 to 1.07) | 0.71 (0.68 to 0.74) | <0.001 |
| **Diabetes mellitus** | | | | | |
| Intervention 1 | Change in intercept | 0.92 (0.76 to 1.11) | 0.77 (0.71 to 0.84) | 1.19 (0.95 to 1.49) | 0.140 |
|  | Change over time | 0.90 (0.87 to 0.94) | 0.89 (0.88 to 0.91) | 1.02 (0.97 to 1.07) | 0.500 |
| Intervention 2 | Change in intercept | 1.08 (0.94 to 1.25) | 0.99 (0.91 to 1.07) | 1.10 (0.92 to 1.31) | 0.308 |
|  | Change over time | 0.98 (0.90 to 1.07) | 1.04 (0.99 to 1.09) | 0.95 (0.85 to 1.05) | 0.290 |
| **Obesity** | | | | | |
| Intervention 1 | Change in intercept | 1.27 (1.12 to 1.45) | 1.02 (0.97 to 1.08) | 1.25 (1.08 to 1.44) | 0.002 |
|  | Change over time | 0.89 (0.86 to 0.92) | 0.90 (0.89 to 0.91) | 0.99 (0.95 to 1.02) | 0.416 |
| Intervention 2 | Change in intercept | 1.41 (1.29 to 1.54) | 0.97 (0.92 to 1.02) | 1.46 (1.31 to 1.61) | <0.001 |
|  | Change over time | 0.85 (0.81 to 0.90) | 1.00 (0.96 to 1.03) | 0.85 (0.80 to 0.91) | <0.001 |
| **Hypertension** | | | | | |
| Intervention 1 | Change in intercept | 1.41 (1.22 to 1.64) | 1.18 (1.13 to 1.24) | 1.20 (1.02 to 1.40) | 0.025 |
|  | Change over time | 0.94 (0.91 to 0.97) | 0.93 (0.92 to 0.94) | 1.01 (0.98 to 1.05) | 0.455 |
| Intervention 2 | Change in intercept | 1.05 (0.91 to 1.22) | 1.04 (0.98 to 1.10) | 1.01 (0.87 to 1.18) | 0.876 |
|  | Change over time | 1.06 (0.97 to 1.15) | 1.13 (1.09 to 1.17) | 0.93 (0.85 to 1.02) | 0.140 |
| **Anti-diabetic medication** | | | | | |
| Intervention 1 | Change in intercept | 0.91 (0.74 to 1.12) | 0.88 (0.81 to 0.95) | 1.04 (0.83 to 1.30) | 0.728 |
|  | Change over time | 0.91 (0.87 to 0.95) | 0.92 (0.91 to 0.94) | 0.99 (0.94 to 1.04) | 0.636 |
| Intervention 2 | Change in intercept | 0.99 (0.86 to 1.15) | 0.99 (0.92 to 1.06) | 1.00 (0.85 to 1.18) | 0.976 |
|  | Change over time | 1.02 (0.94 to 1.11) | 1.00 (0.95 to 1.04) | 1.03 (0.94 to 1.13) | 0.559 |
| **Lipid-modifying medications (including statins)** | | | | | |
| Intervention 1 | Change in intercept | 1.15 (0.99 to 1.33) | 1.04 (0.99 to 1.08) | 1.11 (0.95 to 1.29) | 0.197 |
|  | Change over time | 0.78 (0.75 to 0.81) | 0.76 (0.75 to 0.77) | 1.03 (0.99 to 1.07) | 0.172 |
| Intervention 2 | Change in intercept | 1.06 (0.97 to 1.15) | 0.95 (0.91 to 0.99) | 1.12 (1.02 to 1.23) | 0.023 |
|  | Change over time | 0.98 (0.93 to 1.03) | 1.04 (1.01 to 1.07) | 0.94 (0.89 to 0.99) | 0.031 |

Changes are reported for patients with and without severe mental illness, and the difference between the two groups. SMI, severe mental illness. Intervention 1, 2004 Quality and Outcomes Framework (QOF) indicators. Intervention 2, 2011 QOF indicators.

* p value for difference between groups
