# Supplementary material for: Financial incentives improve recognition but not treatment of cardiovascular risk factors in severe mental illness
Source: PLoS One. 2017 Jun 9;12(6):e0179392. doi: 10.1371/journal.pone.0179392 (PMC5466340; doi:10.1371/journal.pone.0179392)
Supplement: S5 Appendix — (DOCX) [file pone.0179392.s005.docx]

**Appendix S5: Falsification test**

|  | | Odds ratio (95% confidence interval) | | |  |
| --- | --- | --- | --- | --- | --- |
|  | | SMI | Non-SMI | Difference | p value* |
| **Serum cholesterol ≥ 5.0mmol/L** | | | | | |
| Fake intervention 1 (2000) | Change in intercept | 1.33 (1.13 to 1.57) | 1.35 (1.28 to 1.42) | 0.99 (0.83 to 1.17) | 0.815 |
|  | Change over time | 1.05 (0.97 to 1.13) | 1.04 (1.02 to 1.07) | 1.01 (0.93 to 1.09) | 0.696 |
| Fake intervention 2 (2008) | Change in intercept | 1.03 (0.96 to 1.11) | 1.15 (1.12 to 1.19) | 0.89 (0.82 to 0.97) | 0.006 |
|  | Change over time | 0.96 (0.92 to 1.00) | 1.05 (1.03 to 1.07) | 0.91 (0.87 to 0.96) | <0.001 |
| **Diabetes mellitus** | | | | | |
| Fake intervention 1 (2000) | Change in intercept | 1.47 (1.11 to 1.97) | 1.20 (1.05 to 1.37) | 1.09 (0.99 to 1.20) | 0.095 |
|  | Change over time | 1.01 (0.88 to 1.15) | 0.99 (0.93 to 1.06) | 0.99 (0.94 to 1.04) | 0.708 |
| Fake intervention 2 (2008) | Change in intercept | 1.01 (0.84 to 1.22) | 1.09 (0.99 to 1.20) | 0.93 (0.75 to 1.15) | 0.511 |
|  | Change over time | 1.04 (0.94 to 1.15) | 0.99 (0.94 to 1.04) | 1.05 (0.93 to 1.17) | 0.424 |
| **Obesity** | | | | | |
| Fake intervention 1 (2000) | Change in intercept | 0.96 (0.90 to 1.02) | 0.96 (0.94 to 0.98) | 1.00 (0.93 to 1.06) | 0.890 |
|  | Change over time | 0.96 (0.93 to 0.99) | 0.98 (0.97 to 0.99) | 0.98 (0.95 to 1.02) | 0.325 |
| Fake intervention 2 (2008) | Change in intercept | 1.01 (0.95 to 1.07) | 0.97 (0.94 to 1.00) | 1.04 (0.98 to 1.11) | 0.209 |
|  | Change over time | 0.98 (0.95 to 1.01) | 0.96 (0.95 to 0.98) | 1.02 (0.98 to 1.06) | 0.271 |
| **Hypertension** | | | | | |
| Fake intervention 1 (2000) | Change in intercept | 1.40 (1.15 to 1.69) | 1.25 (1.17 to 1.34) | 1.12 (0.91 to 1.37) | 0.282 |
|  | Change over time | 1.04 (0.94 to 1.14) | 1.10 (1.06 to 1.14) | 0.94 (0.86 to 1.04) | 0.243 |
| Fake intervention 2 (2008) | Change in intercept | 0.93 (0.81 to 1.08) | 1.05 (0.99 to 1.12) | 0.89 (0.76 to 1.04) | 0.139 |
|  | Change over time | 0.97 (0.89 to 1.05) | 1.03 (0.99 to 1.06) | 0.94 (0.86 to 1.03) | 0.204 |
| **Anti-diabetic medication** | | | | | |
| Fake intervention 1 (2000) | Change in intercept | 1.39 (1.05 to 1.83) | 1.03 (0.92 to 1.17) | 1.35 (0.99 to 1.82) | 0.055 |
|  | Change over time | 0.97 (0.85 to 1.10) | 0.92 (0.87 to 0.98) | 1.05 (0.90 to 1.21) | 0.552 |
| Fake intervention 2 (2008) | Change in intercept | 1.02 (0.86 to 1.22) | 1.05 (0.96 to 1.15) | 0.97 (0.80 to 1.18) | 0.785 |
|  | Change over time | 1.11 (1.01 to 1.21) | 1.01 (0.96 to 1.06) | 1.10 (0.99 to 1.22) | 0.085 |
| **Lipid-modifying medications (including statins)** | | | | | |
| Fake intervention 1 (2000) | Change in intercept | 0.91 (0.71 to 1.17) | 0.98 (0.90 to 1.06) | 0.93 (0.72 to 1.21) | 0.606 |
|  | Change over time | 0.95 (0.84 to 1.08) | 1.04 (1.00 to 1.08) | 0.91 (0.80 to 1.04) | 0.162 |
| Fake intervention 2 (2008) | Change in intercept | 0.94 (0.86 to 0.97) | 1.01 (0.97 to 1.06) | 0.93 (0.84 to 1.03) | 0.163 |
|  | Change over time | 0.88 (0.83 to 0.93) | 0.95 (0.92 to 0.97) | 0.93 (0.87 to 0.98) | 0.013 |

Changes are reported for patients with and without severe mental illness, and the difference between the two groups. SMI, severe mental illness.

* p value for difference between groups
